# “Academia Is a Very Unforgiving Space”: A Qualitative Study of Challenges Faced by Under-represented Scholars in Biomedical Research

**DOI:** 10.1007/s11606-026-10366-x

**Published:** 2026-04-06

**Authors:** Holly N. Thomas, Marie K. Norman, Hemika Vempalli, Megan Hamm, Gretchen E. White, Natalia E. Morone, Audrey J. Murrell, Doris M. Rubio, Nancy Gauvin

**Affiliations:** 1 Department of Medicine, University of Pittsburgh, Pittsburgh, PA USA; 2 Department of Medicine, Boston University, Boston, MA USA; 3 Department of Business Administration, University of Pittsburgh, Pittsburgh, PA USA; 4 Department of Health and Rehabilitation Sciences, University of Pittsburgh, Pittsburgh, PA USA

**Keywords:** under-represented in medicine (UIM), biomedical research workforce, academic medicine, career barriers, diversity, equity, and inclusion (DEI), mentorship, qualitative research, workforce diversity, early-career investigators, retention in academia

## Abstract

**Background:**

Diversity on biomedical research teams may lead to higher quality research, but some groups remain under-represented (UR) on research teams. Evaluating the challenges that UR scholars in the health sciences face could inform policies that institutions could take to recruit and retain UR scholars.

**Objective:**

We used a large qualitative dataset to examine challenges in pursuing and persisting in research careers among UR postdoctoral and early-career faculty scholars participating in a randomized controlled trial.

**Design:**

We conducted 78 individual qualitative interviews. Most participants held MDs or PhDs and were Black, Hispanic, or biracial. Interviews used a semi-structured guide and were audio-recorded and transcribed. A team of investigators developed an initial codebook based on a subset of interviews, which was iteratively revised by the team. After a final codebook was agreed upon, codes were assigned to all data. Codes were grouped into themes and sub-themes, which were discussed by the team. We present key themes and illustrative quotes.

**Key Results:**

Career challenges reported by UR scholars came from the culture of academia as well as from external factors. Academia culture challenges included demand for productivity and funding, bias and racism, competition and hierarchy, and the “secret rules” to the system. External challenges included financial stress, being an immigrant, and family demands. Responses to challenges could be positive, including making a career pivot, building resilience, finding one’s community, or finding one’s passion. Negative consequences to challenges included burnout, imposter syndrome, and low belongingness.

**Conclusions:**

Challenges faced by UR scholars can be both internal and external to the academy and can induce negative consequences among UR scholars, but some scholars also build resilience and find passion in the face of these challenges. Efforts to improve the culture of academia and to foster positive mentoring could help retain UR scholars in biomedical science.

## BACKGROUND

Diversity on biomedical research teams may lead to higher quality research. Multicultural research teams are associated with greater likelihood of recruiting racially diverse clinical trial participants,^1^ which is important for external validity. Papers with more cultural heterogeneity among the authors are more likely to be published in high-impact journals and are more likely to be cited than papers with more ethnically homogenous authors.^2,3^ More diverse research teams also have higher creativity and satisfaction.^4^

Some groups remain under-represented (UR) in biomedical research. UR in this context is typically defined as Black, Hispanic, Indigenous, and Native Hawaiian/Pacific Islander individuals. These individuals are UR at both the graduate school and post-graduate levels. Only 13% of doctoral graduates in biomedical fields come from UR groups, and only 11% of postdoctoral fellows at academic institutions come from UR groups.^5,6^ UR postdoctoral trainees are much less likely to transition into faculty positions compared to trainees who are not from UR groups.^5^ Within academic medical centers, UR faculty members are half as likely to be promoted to full professors and half as likely to remain in academic medicine over time compared to White faculty members, even when adjusting for other factors.^6^

Prior research has examined the challenges that UR scholars in the health sciences face. These studies have highlighted the experience of bias, low belongingness,^7–9^ and stress regarding the need for grant funding as key challenges.^10,11^ This prior research largely focuses on undergraduate students; less is known about postdoctoral scholars and early-career faculty members. Moreover, many of these prior studies are quantitative, which can miss important themes regarding an under-studied subject; the qualitative studies that do exist have smaller samples. Identifying challenges that UR postdoctoral scholars and early-career faculty members in the health sciences face could inform policies at the academic institutional, state, and federal levels to recruit and retain these scholars. In this study, we aimed to fill this research gap by using a large qualitative dataset to examine career challenges among UR postdoctoral and early-career scholars participating in a randomized controlled trial.

## METHODS

Building Up was a cluster-randomized controlled trial conducted at 25 academic institutions comparing two interventions seeking to support early-career UR scholars. Participants in one arm received a novel intervention with mentorship from near-peer (mid-career) mentors, networking opportunities, and coursework in manuscript and grant writing. In the other arm, participants received usual mentoring, networking, and coursework as offered by their institutions. Both groups attended a monthly leadership webinar. The details of Building Up have been previously described.^12–17^ This study was approved by the Institutional Review Board of the University of Pittsburgh, and participants provided informed consent. Clinical trial number: not applicable.

### Participants

Participants had to (1) be from a UR racial or ethnic group, have a disability, be from a disadvantaged background, or be a woman;^18^ (2) hold a terminal degree (MD, PhD, etc.); (3) be a postdoctoral fellow or early-career faculty; and (4) be committed to a career in research. For recruitment, each institution was first recruited to be a site (with a champion at each site), and then the champion recruited participants at their institution for the trial.

### Qualitative Interviews

Building Up participants were randomly selected for participation in qualitative interviews, with randomization being stratified by site. The initial goal was to conduct 100 interviews. Thematic saturation was reached after 78 interviews. Two experienced interviewers at the University of Pittsburgh’s Center for Biostatistics and Qualitative Methodology conducted one-hour semi-structured interviews over videoconferencing software with scholars in both arms of the trial at trial completion. Many questions focused on participants’ experiences with the programmatic elements of the Building Up intervention. For this analysis, we focused on the participants’ responses to one question: “Can you tell me about the biggest challenge in your career so far?”

Interviews were audio-recorded and transcribed with potentially identifying information omitted. We used an inductive thematic analysis approach to coding.^19^ The lead author read through all interviewee’s responses and presented initial themes to other members of the authorship team. The team developed a codebook and the lead author began coding a subset of 10 interviews. The lead author has over 10 years of experience in conducting and analyzing qualitative interviews, with numerous peer-reviewed qualitative publications. The team met again and discussed the codes applied thus far and themes that were emerging. The codebook was refined based on this discussion. The lead author then proceeded to assign codes to all data. Once all data were coded, the team grouped codes into sub-themes and larger themes.

### Reflexivity Statement

The authors represent a variety of racial and ethnic groups, including White, South Asian, Hispanic, and Black. All authors are female. All authors are part of academia at different levels, ranging from post-doctoral up to full professor. The authorship team includes medical doctors, administrators, educators, and anthropologists. Many of the authors have participated in prior research on UR groups in biomedical research.

### Demographic Information

Participants completed a pre-intervention survey that included demographic information regarding birth year, race/ethnicity, degree type, career stage, whether they were raised by an adult with a bachelor’s degree, and whether they had a disability. This demographic information was matched to interview data.

## RESULTS

The Building Up Cohort has been previously described.^14,20^ Briefly, 80% of the 224 participants in the trial were female, 59% held a PhD as their terminal degree, 53% were early-career faculty, 34% identified as Hispanic, and 33% identified as non-Hispanic Black. Regarding the 78 interview participants, most participants were female (79.0%) and held a PhD as their terminal degree (56.8%). Most (56.9%) were currently in a faculty position. Regarding race/ethnicity, 48.5% were Hispanic, 35.3% were Black, 7.4% were Asian, 5.9% were Middle Eastern or North African, and 2.9% were Native American. The parents of most participants had completed a bachelor’s degree (67.1%), and most of the participants did not report a disability (94.4%) (Table 1).

**Table 1:** Demographic Data for Participants in Qualitative Interviews from Building Up, *N* (%)

Age (mean, SD)	39.1 (6.0)
Disability	
No	67 (94.4)
Yes	4 (5.6)
Gender	
Female	60 (79.0)
Male	15 (19.7)
Transgender/non-binary	1 (1.3)
Degree	
MD	27 (36.5)
PhD	42 (56.8)
Other	5 (6.8)
Parent completed bachelor’s degree	
No	25 (32.9)
Yes	51 (67.1)
Position	
PhD	1 (1.5)
Postdoc	27 (41.5)
Faculty	37 (56.9)
Race/ethnicity	
Asian	5 (7.4)
Black	24 (35.3)
Hispanic	33 (48.5)
Middle Eastern/North African	4 (5.9)
Native American	2 (2.9)

**Figure 1:**
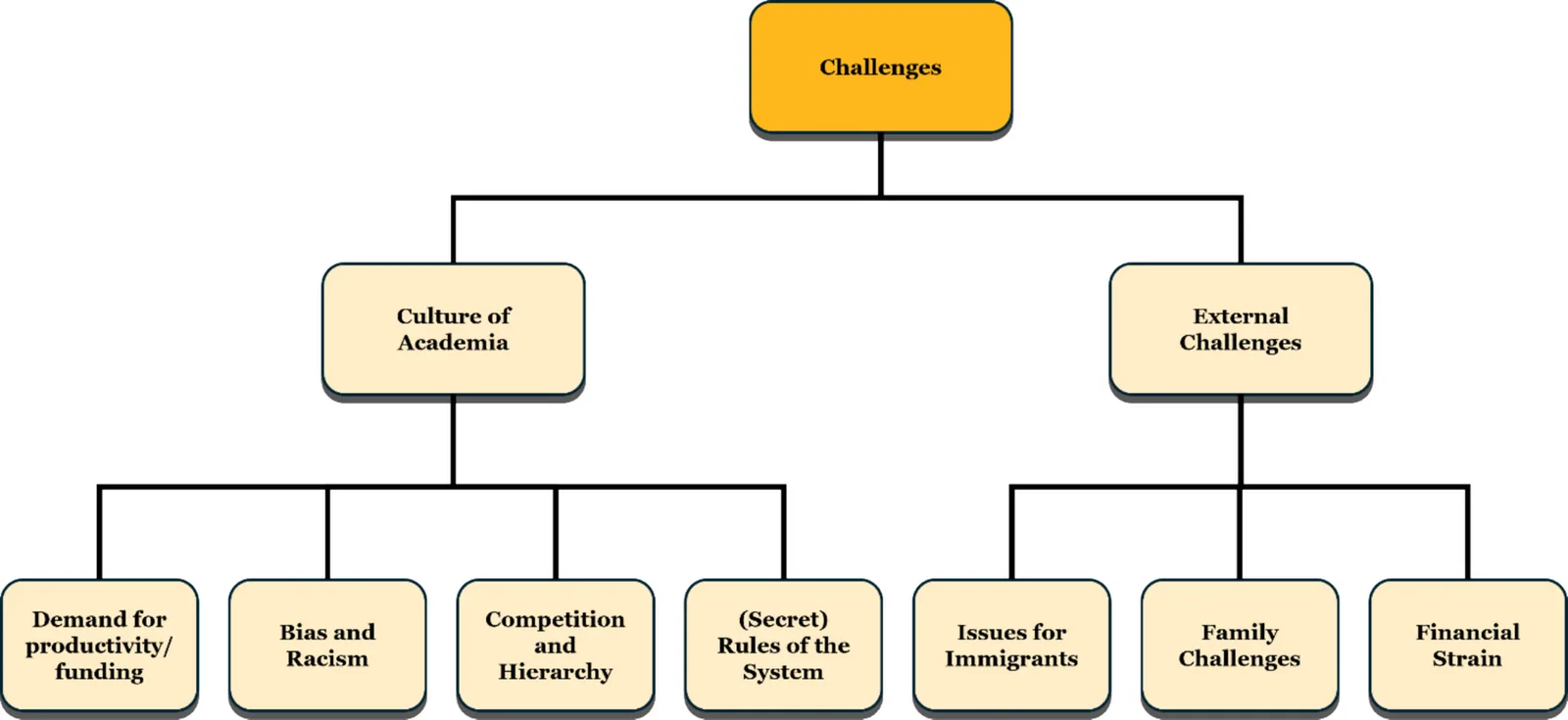
Challenges faced by under-represented scholars in biomedical research.

Thematic analysis revealed key themes around the career challenges of early-career UR investigators. Challenges discussed came from the culture of academia and external factors (Fig. 1). Culture of academia challenges included demand for productivity and funding, bias and racism, the competitive and hierarchical culture, and a sense that there are secret rules to the system.

### Culture of Academia Challenges

#### Demand for Productivity and Funding

Scholars discussed the stress brought on by academia’s demand for constant productivity and grant funding. They discussed feeling that regardless of how many papers or grants they submitted, there was always a demand for more.

In academic medicine there is never a period of harvest. There’s never a period where you get to reap what you sow and rest. You are constantly attempting to sow more, and so as soon as you finish one manuscript and it’s published, now it’s on to the next one. Your grant is finished, now you get to apply for the next R01. You are constantly on this hamster wheel of attempting to be academically productive, and you never have a period of just rest.—Black woman

In addition, participants felt that coming from a marginalized background can create even more pressure than the culture of academia alone. She shared:

I keep pushing myself harder, because of my being double minority, I feel the need to go above and beyond. I can’t just be average to achieve my goals here. It puts so much pressure on me, to the point that I have reached burnout more than once already.—Hispanic woman

The constant demand for productivity may not be unique to UR scholars, but UR scholars may feel its impacts more. UR scholars may feel the need to over-perform to overcome institutional and systemic barriers to academic progress.

The demand for productivity was often related to the need for grant funding. Scholars noted that navigating soft money positions could be stressful, and they feared that if funding is lost, they will not be able to continue in science. One participant highlighted how their personal history shaped their perception of financial stability:

The soft money environment is very stressful for me. I grew up working class and the money feels like real money to me. People who have been doing soft money for a long time, it doesn’t really feel like real money, it just feels like play money… Everybody that I know who does this, we’re all stressing stressing stressing stressing about staying fully funded.—Middle Eastern or North African woman

Many scholars discussed growing up in a financial situation that can lead to persistent worries about poverty as an adult, even when the immediate threat of poverty no longer exists, heightening the impact of worries about grant funding.

#### Bias and Racism

Scholars talked about experiences with both overt and implicit bias based in racism, sexism, and xenophobia. These experiences could be brought about by the participants’ skin color, accent when speaking, or other cultural differences. Black women were particularly affected.

When people see me in the lab, [I’m] always being thought of as a technician or a student or even housekeeping. Nothing’s wrong with any of those positions, and I’m not saying that I’m better than any of them at all, but I’ve worked for my degree. And those assumptions are not placed on my coworkers in the same space. It reinforces the imposter syndrome that I struggle with. I’m an African American woman, I have a child, all these things that while openly we say is OK and fine, I’m not sure.—Black woman

This scholar describes how microaggressions experienced in the institution fed into imposter syndrome. She notes that while the institution says it supports women, Black individuals, and parents, it is difficult for her to trust, and she wonders if these are the true values of the institution.

[My biggest challenge is] proving my worth so that I’m able to pursue promotions or raises. This is a university-wide problem. This is the main reason that most of the minority females that I’ve worked with… have left [name of institution] is because they set these goals, you work with your mentors, you work with the department, and if I meet these things, we can discuss promotion… I’ve had a peer that left in the last year. She got a K award… And they told her, if you do this we will promote you to faculty. And they just didn’t… So, she left [name of Institution] and returned her grant funding back to NIH. She’s not the first example that I have of this… My fear is that I’m being pulled along in that same fashion... It just seems to be here a little bit, one more this, one more that, show me this, and then we’ll promote you. But in all actuality the intention is not truly there.—Black woman

This scholar notes that female and UR faculty members have a more difficult time achieving pay raises or promotions, have difficulty trusting promises from the institution, and perceive a need to work exceedingly hard to achieve recognition.

#### Competition and Hierarchy

Many scholars, especially women, reported difficulty managing the competitive and hierarchical culture of academia, noting that academic medicine often involves big egos. Some scholars noted that it was important to them to be motivated in research via a desire to give back to one’s community, rather than to grow one’s ego.

[My biggest challenge is] just finding my voice. People like me, we’re so humble and we’re servant leaders and I’m trying to learn to be more of a, quote unquote, ‘mainstream leader,’ at the same time not perpetuate that leadership style, because that’s what’s gotten us into this mess in the first place. How do we become leaders that are humble and that actually invite everyone to the table… How do we change this hierarchy approach that we’ve constantly given every day to a more horizontal way of relating each other and humanizing. At the end of the day, I’m tired of being in a system that just dehumanizes people and dehumanizes me. So, every day I try to think of ways to humanize that process and not lose myself.—Hispanic/Latina woman

Scholars described struggling to stay true to their core values, especially when they desire to have a less hierarchical leadership structure yet feel the need to participate in the hierarchy to climb the ladder. Adjusting to this culture can be even more difficult for scholars who grew up outside the USA.

The biggest challenge has been overcoming the culture shock. I lived most of my life in [country]. My training was done in [country], and so my biggest challenge since I moved here was to learn how science is made here in the US. To see how much more competitive the field is here.—White Hispanic/Latina woman

#### (Secret) Rules to the System

Scholars discussed that there were protocols within academia that were not always readily apparent, especially for someone who might be the first in their family to enter this environment.

It seems like there is another way to do it here [in the United States]. Because [of] the lack of mentor[ship], I normally do things as they make sense in my head… and maybe I am escaping protocols, in the sense of, ‘if you want to talk to [a] mentor maybe draft an email like this, make a first contact like this’, something more formal… I feel like not all people are open to diversity. That’s our reality. So, you don’t feel really welcome in every place that you go.—White Hispanic/Latino man

This scholar notes that having mentorship is a key part of navigating these “secret” rules, tying these rules back to academia’s unwillingness to be truly open to differences. Others who are already understand the culture of academia may be less forgiving to someone who is newer to the culture. In contrast, the following scholar discusses how proactive, positive mentoring allowed her to understand the process for progressing in an academic career.

I didn’t really know how to navigate the academic careers… I had no idea what I was doing… But thankfully I had mentors who helped me through. For example, I was finishing my PhD, and I didn’t have a postdoc lined up. And so, my mentoring team encouraged me to reach out to people that I knew through conferences and things like that. Just start cold-calling people. If I didn’t have that mentoring team, I sure as heck wouldn’t have had the nerve to do something like that.—White woman

Some scholars struggled with the balance between playing within the rules of the system versus trying to push back on a system they perceived as unfair to improve things for themselves and future generations.

### External Challenges

External factors discussed included financial stress and poverty, issues related to being an immigrant, and family demands such as parenting.

#### Financial Stress and Poverty

Scholars in our study reported feeling financial stress in their personal lives. Some scholars mentioned how, unlike some of their peers, they had to work while obtaining degrees, which added to their stress.

The financial aspect has been extremely tough, because pursuing science—the putting off financial reward for the amount of energy and effort for folks who are coming from a place of lack is definitely tough. Being broke and saying okay, I’m gonna continue to do this. Moved out the projects and now I’m in an apartment, but I’m thirty-some-odd years old and I can only afford an apartment that’s not even a good apartment, is not a reasonable thing for people to accept.—Black man

This scholar notes that these challenges can be compounded by coming from a disadvantaged background. Even an “upgrade” from the standard of living they may have experienced earlier in life can seem not worth all the energy and effort that they are expected to put forth.

#### Being an Immigrant

Many of the scholars in Building Up immigrated to the USA to pursue careers in science. These scholars faced unique challenges, including language difficulties, a new culture in both their personal and professional lives, and maintaining a visa. Even when they have mastered English as a second language, they may still experience negative responses from others due to an accent. The stakes of success or failure are often higher for individuals who have immigrated to pursue science; the loss of a position and in turn a work visa can mean returning to a country that might be impoverished or dangerous or could mean relinquishing the ability to do the work they have trained for years to do.

My first postdoc position was terminated suddenly, and I had to scramble within a relatively short time to find a new position. I require a visa to work in the United States, so finding a position that would sponsor that was very stressful. Thankfully, I was able to reach out to a network that provided me with the position that was able to patch me through to avoid having to leave the United States or leave the work that was here. Adapting to that, working out the logistics, [and] coordinating moves around the country on a very limited budget was very stressful.—White Hispanic/Latino man

#### Family Demands

Scholars discussed difficulties with work-life balance, often centered around roles as parents. These concerns were more often cited by female participants than by male participants in Building Up.

My biggest challenge. Probably imposter syndrome… In my lab I’m the only one who has kids. I can’t always just stay at lab and push through things, or work on the weekends… It makes me feel like I’m less of a scientist sometimes. I question, am I working hard enough, do I deserve to be here, am I smart enough?—Black woman

This scholar discusses how being a parent feeds into imposter syndrome. However, some participants mentioned how their children were a source of inspiration and energy.

### Consequences of Challenges

**Figure 2:**
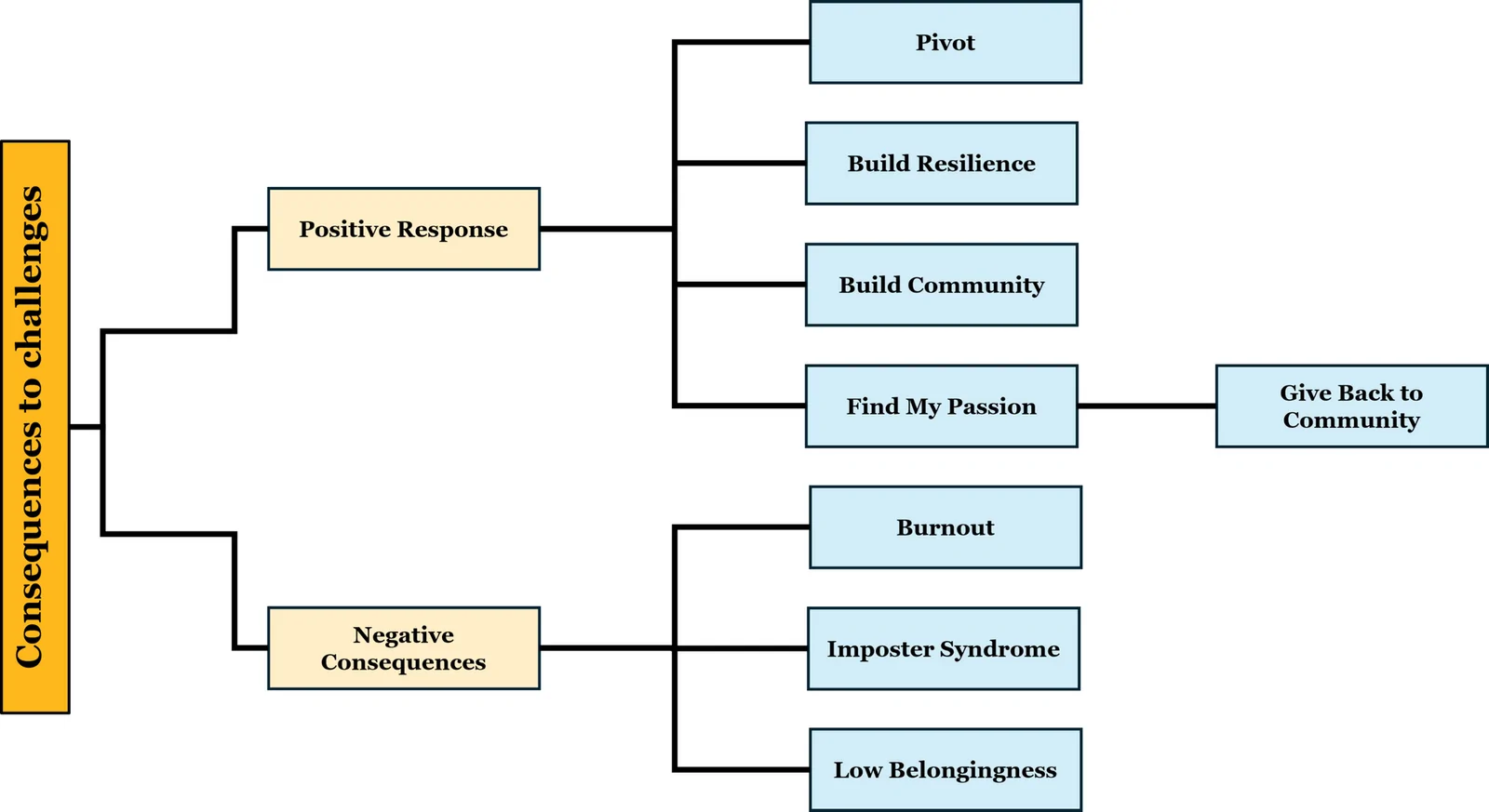
Consequences of challenges faced by under-represented scholars in biomedical research.

Challenges resulted in both positive and negative consequences (Figs. 1, 2). Positive responses included making a career pivot, building resilience, finding one’s community, and finding one’s passion. Negative consequences discussed by scholars included burnout, imposter syndrome, and feelings of low belongingness.

#### Positive Consequences

Participants mentioned several positive consequences or results of challenges, including (1) Pivoting, (2) Building Resilience, (3) Finding Community, and (4) Finding Their Passion. Each of these positive consequences will be discussed in turn.

##### Pivoting

Many scholars discussed having to make a transition due to challenges, often achieved with the assistance of supportive mentors; responding with a pivot as opposed to characterizing it as a failure allowed scholars to continue working towards their goals.

The biggest thing that my mentors and I did was pivot. We realized that there wasn’t anything we could do about the current situation, and we tried to make the best out of it. We looked for other opportunities outside of being hired on as faculty, and other ways to have my salary funded… it ended up working out great because I got ingratiated into another department and developed mentors in that department, which was actually really fantastic. It was taking the situation, evaluating what we could do and could accomplish, and going full speed ahead in that direction rather than lamenting what we could have done.—White woman

##### Building Resilience

Many scholars discussed that, while failure is difficult, it builds resilience, which is essential for a career in research. Some noted that rejection had a stronger negative impact on them because of their minority status, but others discussed how important it is to continue to “keep your head up” and “let your light shine” in the face of rejection.

I would say [my biggest success has been] getting to where I am despite all I’ve been through. I’ve experienced learning disabilities, leading to me not finishing a medical curriculum. That’s still something I think about. That’s not an easy thing to get over. My resilience is my biggest accomplishment.—Hispanic/Latino man

##### Finding Community

Scholars discussed that building community was an important way to handle challenges. Some discussed how finding other UR scholars served as a buffer.

At the end of the day, it’s those moments, those relationships with the people, that you have. It’s your … family and your community. Those are the things that sustained me moving forward.—Middle Eastern or North African woman

##### Finding My Passion

Scholars also noted that challenges could point them on a new path that allowed them to discover their true passion. They noted that setting out in a field that others may not consider mainstream or academically fruitful is difficult. Some noted that if they were focused on equity or justice, these topics may not seem as attractive or popular to other scholars or journals.

I wanted to do global oncology in adolescents for many years and was told that it wasn’t really a thing… So I just started doing things on the side… as a passion project. Then I decided to go for it and apply for a career development award, which I received. I feel like that’s my biggest success. Not so much because these awards are prestigious, but more that, even though I wasn’t getting all of the support that I would have liked, I stuck with it, because it was… really important to me…—Hispanic/Latina woman

Her passion for this topic was not just because it was interesting, but because she felt it would make a positive difference for underserved populations. This motivation sustained the scholar in pursuing this passion, even in the face of discouragement. Likewise, many scholars discussed how pursuing their passion was important to them because it allowed them to give back to the communities from which they came.

#### Negative Consequences

Participants mentioned several negative consequences of challenges, including (1) Burnout, (2) Imposter Syndrome, and (3) Low Belongingness. Each consequence will be discussed in turn.

##### Burnout

Many scholars discussed experiencing burnout, even though they are relatively early in their careers. This burnout was often perpetuated by the culture of academia, described above. For example, burnout can result from feeling the need to over-perform to challenge inherent bias or from feeling like those in power do not fully support the type of work that one is doing.

[My biggest challenge is] frustration at seeing how unwilling some people are to accept a lot of the problems that academia has on a systemic level… this constant sense of burnout and frustration at trying to move things in ways that people and leadership don’t necessarily think are needed, or hitting brick walls, trying to sell people on the idea that academia needs to be inclusive and equitable and a supportive space of growth, not because it leads to better science, but because people who have been harmed by and actively minoritized by the system deserve better, period.—Hispanic Latine transgender woman/non-binary individual

##### Imposter Syndrome

Experiencing imposter syndrome was commonly discussed as a response to challenges. Participants discussed how the culture of academia can fuel imposter syndrome.

I have this mindset of imposter syndrome and constant feeling like I can’t say no, and I have to prove myself, and I need to be able to do it all. I’m reaching a point where it’s not sustainable. It’s going to take a reframing of my mindset… It is okay to take a step back and figure out which commitments do you need and which do you not. … It’s a challenge that I’m actively working through… My mentor said to me, ‘You don’t have to impress others anymore, you’ve done the work, people know that you can do this and you’re going to be a success, because you already *are* a success.’ I’ve never thought of myself like that; in my mind I still…envision this struggling medical student that’s the first in her family and doesn’t know how the process of applying to medical school works… It will require a period of time to relearn how I should be thinking about myself.—White and Indigenous (American Indian/Alaska Native) woman

This scholar also notes how a disadvantaged background can make imposter syndrome more difficult to overcome, but how a good mentor can help a scholar combat imposter syndrome. Likewise, the following scholar reflects on how positive and negative mentoring experiences can affect imposter syndrome.

Mentors have helped me feel like the imposter as well as helped me get over feeling like the imposter… The strongest feelings of ‘I don’t belong here’… have probably come from pressure from mentors… But also, feeling like I do belong has come from having a collection of multiple mentors and being more strategic about how I listen to certain mentors… Academia is a very unforgiving space, so there are just certain mentors who I should listen to differently, and that’s been helpful.—Black woman

##### Low Belongingness

Scholars discussed feelings of low belongingness, such as being “the only one who looks like me” in academic spaces. Participants discussed how being the only one from a different culture in a particular lab can stymie career progress.

The challenge was the abuse that I put up with from my advisor, verbal abuse. There were some racial issues. It was a [racial group] laboratory and I was the only one who was not [racial group]… I was definitely looked over for opportunities. I couldn’t quite get plugged in. My advisor was not helpful, to put it nicely… It was not an easy thing to be a postdoc in that lab for me, for five years.—Hispanic/Latino man

The mentors and advisors that were working with this scholar did not provide him the support he needed in this cross-cultural lab situation.

## DISCUSSION

In this large set of qualitative interviews with early-career UR scholars, we found that scholars faced many challenges, some emanating from the culture of academia and others from external factors. Some of our findings closely align with prior work. The expectation for high productivity, hypercompetitive environments, concerns about funding, feeling the need to over-perform, and low belongingness have been discussed in prior research with UR scholars.^9,21–23^

Our work also aligns with some of the research on challenges faced by women in academic medicine and science. Women are much less likely than men to achieve full professorship and leadership positions in academic medicine, even when controlling for other factors.^24–27^ Similar to feelings of low belongingness and “not looking like a scientist” endorsed by participants in our study, women are sometimes perceived to lack the qualities needed to be successful scientists.^28^ Prior research has also discussed the challenges of imposter syndrome, which affects women in science more intensely than men.^29^ Like some scholars in our study, prior research has documented family responsibilities as being a barrier to progress for women in medicine.^24,30^

Some unique themes that our current study uncovered include more explicitly calling out negative experiences as rooted in racial bias and discussing the importance of pivoting when challenges arise. One theme discussed in prior research that was not discussed by participants in our study was a “minority tax,” the idea that UR scholars are often called upon to participate in diversity and equity committees or to mentor early-career UR scholars. In contrast, participants in the current study specifically discussed mentoring more junior UR scholars as “filling their cup.”

Other novel findings that have not been extensively explored in prior work include the challenges of adapting to the “secret” rules of the system, poverty, and being an immigrant. Scholars discussed the challenges of navigating protocols—such as “cold e-mailing” or asking for sponsorship—and feeling confused and stymied. Non-UR scholars may have more experience navigating professional spaces similar to academic medicine due to prior exposure. Proactive mentoring that pulls back the curtain on these secret rules can serve as a buffer for UR scholars. Regarding poverty, chronic stress related to financial strain may have significant negative impacts on UR scholars’ academic productivity. Our work reveals that these financial challenges can be persistent, even at the faculty level. Programs to support early-career scholars financially could offset some of these concerns. Finally, little work has documented challenges related to the immigrant experience. Immigrants may have to navigate the challenges of not only “looking different,” but also “sounding different.” Scholars may be less inclined to give input at meetings and as a result may miss opportunities; in order to buffer against this, supportive mentors could encourage input from UR scholars. Immigrant scholars also discussed worrying about losing a work visa, making the stakes of success in academia that much higher. Current political changes regarding non-US-born researchers at US universities mean these concerns are likely even more acute than when these interviews were conducted.

While some of the challenges discussed are not unique to UR scholars, they may affect UR scholars more deeply. With regard to funding pressure, Black scientists are less likely to receive National Institutes of Health (NIH) funding compared to White scientists, even when controlling for other factors.^31,32^ While competition for grant money is a stressor for all researchers, the impact may be stronger for UR scholars. With regard to financial concerns, prior studies document that Black women physicians report greater medical debt compared to their White women counterparts; as a result, financial worries about maintaining a faculty position may be more acute.^33^ These scholars may be less likely to have family funds or other resources to fall back on if they lose their current position.^6^ Prior research also describes how the perceived cost of failure is higher in UR scholars than their counterparts.^34^ Privilege may serve as a buffer against these stressors among White men. One study compared White able-bodied heterosexual men to 31 other intersectional groups regarding advancement in scientific careers. The men experienced more social inclusion, more professional respect, more career opportunities, higher salaries, and higher intent to persist in science than any of the other 31 groups.^35^

Imposter syndrome and burnout, both discussed by participants in our study, are often framed as conditions that affect the individual, and solutions are often framed at the individual level. For example, institutions may offer psychological counseling resources to support scholars. However, our findings show that these conditions are often exacerbated by key characteristics of academic culture. These systemic issues require systemic, in addition to individual, solutions.

Our work also uniquely highlights the positive responses that UR scholars can develop in response to challenges, such as pivoting or building resilience. To employ these responses, however, scholars require a supportive mentorship team and community. A supportive mentor can help scholars devise the best strategy when challenges arise and may be aware of alternative pathways to achieve goals of which UR scholars are not aware. A supportive community, whether through work peers, family, or friends outside of academia, can help scholars feel strong and valued regardless of what is happening in their career.

This work highlights the importance of supporting not just diversity, but also equity and inclusion. UR scholars discussed feelings of low belongingness. To retain UR scholars and allow them to perform at their highest levels, institutions could enact programs to support equity and inclusion, including supporting UR scholars pursuing research that serves the interests of their own communities.^36^ However, this is challenging given the culture of the current US government administration, which views such programs as discriminatory.

Scholars discussed how mentoring can either contribute or help them overcome challenges. High-quality mentoring acts as a buffer to the negative impacts of racism and discrimination,^37^ and mentoring that addresses diversity and inclusion head-on is key.^12,16^ Mentoring that includes identity work can be particularly powerful.^37,38^ Institutions should connect scholars with mentors that can help them navigate the challenges of academic culture and create channels for scholars to navigate problems that may arise with their mentors. These channels could include academic advisors with diversity, equity, and inclusion expertise, anonymous avenues to report issues of microaggressions or bias, or opportunities for near-peer or peer-to-peer mentoring. Academic institutions should also recognize and celebrate mentors who guide UR scholars to success. Again, enacting such programs may be difficult given the current political climate.

This analysis has limitations. The majority of coding was performed by one investigator, although the codebook was developed and reviewed by a team of investigators, and other investigators reviewed a subset of coded interviews. The participants are heterogeneous, representing a wide variety of identities with regard to race, gender, degree, and other characteristics. However, they do all have in common that they are from groups that are UR in biomedical research and therefore may have many shared experiences and themes. Accepting that people may have many intersecting identities, rather than analyzing qualitative research based on only one identity at a time, may better represent the lived experiences of participants. The interviews were conducted within the context of a randomized controlled trial for UR individuals; therefore, the conduct of the trial itself may have influenced the responses of the participants to questions.

Our findings are timely, given current changes in academic medicine. There are ongoing political challenges to diversity, equity, and inclusion programs within universities, as well as new restrictions on federal grants for UR researchers or research that focuses on health equity. Some of the challenges that scholars discussed in this study may worsen given these changes. If creating and maintaining a diverse research workforce is to continue to be a goal for academic medicine, institutions will have to develop creative solutions to mitigate the expected negative effects.

Despite the challenges identified by early-career UR scholars in our study, several positive themes also emerged. Participants expressed a deep commitment to their research and a strong desire to contribute meaningfully to their communities, often viewing their work as a source of purpose. Many scholars described mentoring relationships, especially those with mentors who shared similar backgrounds or values, as transformative and energizing. Peer and near-peer support networks also played a critical role in fostering resilience and a sense of belongingness. Importantly, scholars found strength in their ability to adapt and pivot in the face of systemic barriers, demonstrating both resourcefulness and determination. These findings suggest that, when adequately supported, UR scholars bring a wealth of passion, innovation, and community-minded leadership to academic medicine—underscoring the potential benefits of investing in equity-focused structures that enable them to thrive.

## Data Availability

De-identified data from this study can be requested from the lead author in coordination with the lead author's academic institution.
